# Whole-Genome Analysis of Clinical Vibrio cholerae O1 in Kolkata, India, and Dhaka, Bangladesh, Reveals Two Lineages of Circulating Strains, Indicating Variation in Genomic Attributes

**DOI:** 10.1128/mBio.01227-20

**Published:** 2020-11-10

**Authors:** Daichi Morita, Masatomo Morita, Munirul Alam, Asish K. Mukhopadhyay, Fatema-tuz Johura, Marzia Sultana, Shirajum Monira, Niyaz Ahmed, Goutam Chowdhury, Shanta Dutta, Thandavarayan Ramamurthy, Prosenjit Samanta, Eizo Takahashi, Keinosuke Okamoto, Hidemasa Izumiya, Makoto Ohnishi

**Affiliations:** a Collaborative Research Center of Okayama University for Infectious Diseases in India, Kolkata, India; b Department of Bacteriology I, National Institute of Infectious Diseases, Tokyo, Japan; c icddr,b - International Center for Diarrheal Diseases Research, Bangladesh, Dhaka, Bangladesh; d ICMR—National Institute of Cholera and Enteric Diseases, Kolkata, India; e Department of Biotechnology and Bioinformatics, University of Hyderabad, Hyderabad, India; Mass General Hospital

**Keywords:** genomics, lineage, phylogenetic analysis, whole-genome sequencing

## Abstract

Vibrio cholerae serogroup O1 is responsible for epidemic and pandemic cholera and remains a global public health threat. This organism has been well established as a resident flora of the aquatic environment that alters its phenotypic and genotypic attributes for better adaptation to the environment. To reveal the diversity of clinical isolates of V. cholerae O1 in the Bay of Bengal, we performed whole-genome sequencing of isolates from Kolkata, India, and Dhaka, Bangladesh, collected between 2009 and 2016. Comparison with global isolates by phylogenetic analysis placed the current isolates in two Asian lineages, with lineages 1 and 2 predominant in Dhaka and Kolkata, respectively. Each lineage possessed different genetic traits in the cholera toxin B subunit gene, *Vibrio* seventh pandemic island II, integrative and conjugative element, and antibiotic-resistant genes. Thus, although recent global transmission of V. cholerae O1 from South Asia has been attributed only to isolates of lineage 2, another distinct lineage exists in Bengal.

## Abstract (precis)

Cholera continues to be a global concern, as large epidemics have occurred recently in Haiti, Yemen, and countries of sub-Saharan Africa. A single lineage of Vibrio cholerae O1 has been considered to be introduced into these regions from South Asia and to cause the spread of cholera. Using genomic epidemiology, we showed that two distinct lineages exist in Bengal, one of which is linked to the global lineage. The other lineage was found only in Iran, Iraq, and countries in Asia and differed from the global lineage regarding cholera toxin variant and drug resistance profile. Therefore, the potential transmission of this lineage to other regions would likely cause worldwide cholera spread and may result in this lineage replacing the current global lineage.

## INTRODUCTION

Cholera, which constitutes a disease of acute severe diarrhea, remains a major public health problem in developing countries. Historically, cholera pandemics have been recorded seven times since 1817, with the seventh cholera pandemic still ongoing since 1961 (1). Recently, serious country-wide cholera epidemics have been reported in both Haiti and Yemen (2, 3). In Haiti, no cholera had been reported until after the earthquake in 2010, at which time cholera spread across the country and became endemic. Nearly 800,000 Haitians have been afflicted by cholera, and over 9,000 have died (4). In comparison, the cholera outbreak in Yemen began in October 2016, resulting in Yemen being the first country on record to report more than one million suspected cases in a single year (5).

Vibrio cholerae comprises over 200 serogroups based on their somatic O antigens, among which serogroup O1 and O139 strains cause epidemic cholera (6). The O1 serogroup is divided into two biotypes, classical and El Tor. Notably, the first six pandemics appear to have been caused by the O1 classical biotype, whereas the El Tor biotype constitutes the causative agent in the seventh pandemic. Moreover, although previously cholera was caused only by biotype O1, in 1992 a new serogroup of V. cholerae, O139, was identified as the cause of epidemic cholera in India and Bangladesh (7).

In addition, several variants of the El Tor biotype have emerged and replaced the typical El Tor (8–12). Representative single nucleotide variations (SNVs) in such variants are found in genes for the cholera toxin B subunit (*ctxB*), major subunit of toxin-coregulated pilus (*tcpA*), multifunctional, autoprocessing repeats-in-toxin toxin (*rtxA*), and *carR*, which encodes the response regulator involved in the resistance phenotype against polymyxin B. For example, early in the seventh pandemic, the typical El Tor isolate possessed *ctxB3*, following which an El Tor variant harboring *ctxB1* emerged and disseminated. More recently, isolates possessing *ctxB7* were detected in the Haiti and Yemen outbreaks. Along with the shift of the *ctxB* genotype from *ctxB3* to *ctxB1*, V. cholerae O1 El Tor acquired the integrative and conjugative element (ICE) of the SXT/R391 family via horizontal transfer. ICE contains several antibiotic resistance genes, including those for sulfamethoxazole and trimethoprim (together often abbreviated as SXT), which had previously been used in the treatment of cholera. Therefore, the current V. cholerae O1 generally exhibits multidrug resistance. There are four pathogenicity islands in the epidemic V. cholerae O1 genome, which are *Vibrio* pathogenicity island 1 (VPI-1), VPI-2, *Vibrio* seventh pandemic island I (VSP-I), and VSP-II. It has also been reported that VSP-II had genetic heterogeneity in El Tor strains, and their characteristics were highly related to the *ctxB* genotype (13–17).

Recently, whole-genome sequencing analysis of V. cholerae strains has provided fresh insight regarding the sites of strain emergence and worldwide spread. Several recent articles based on whole-genome sequencing have depicted South Asia as serving as a cradle of all epidemic and pandemic strains that lead to waves of cholera outbreaks worldwide (9, 12, 18, 19). In particular, the Bay of Bengal area in South Asia is known as a zone for cholera endemicity, and it is considered that this region constitutes a hot spot for the emerging epidemic strains. Thus, changes in epidemic V. cholerae O1 in the Bay of Bengal area may influence the globally prevalent V. cholerae. To understand the phylogeny of V. cholerae O1 in Bengal, we performed whole-genome sequencing of 125 clinical V. cholerae O1 isolates in Kolkata, India, and Dhaka, Bangladesh, collected from 2009 to 2016. The isolates were divided into two lineages by phylogenetic analysis and exhibited different genetic traits of *ctxB*, ICE, and VSP-II in accordance with each lineage. The population structure of the dominant lineage differed between Kolkata and Dhaka, although isolates from both lineages were detected in each region. Therefore, two distinct lineages which likely constitute candidates for generating new cholera pandemics exist in Bengal and represent a global threat for not only international but also intercontinental transmission.

## RESULTS

### Phylogenetics and phylodynamics of V. cholerae O1 in Kolkata and Dhaka.

**FIG 1:**
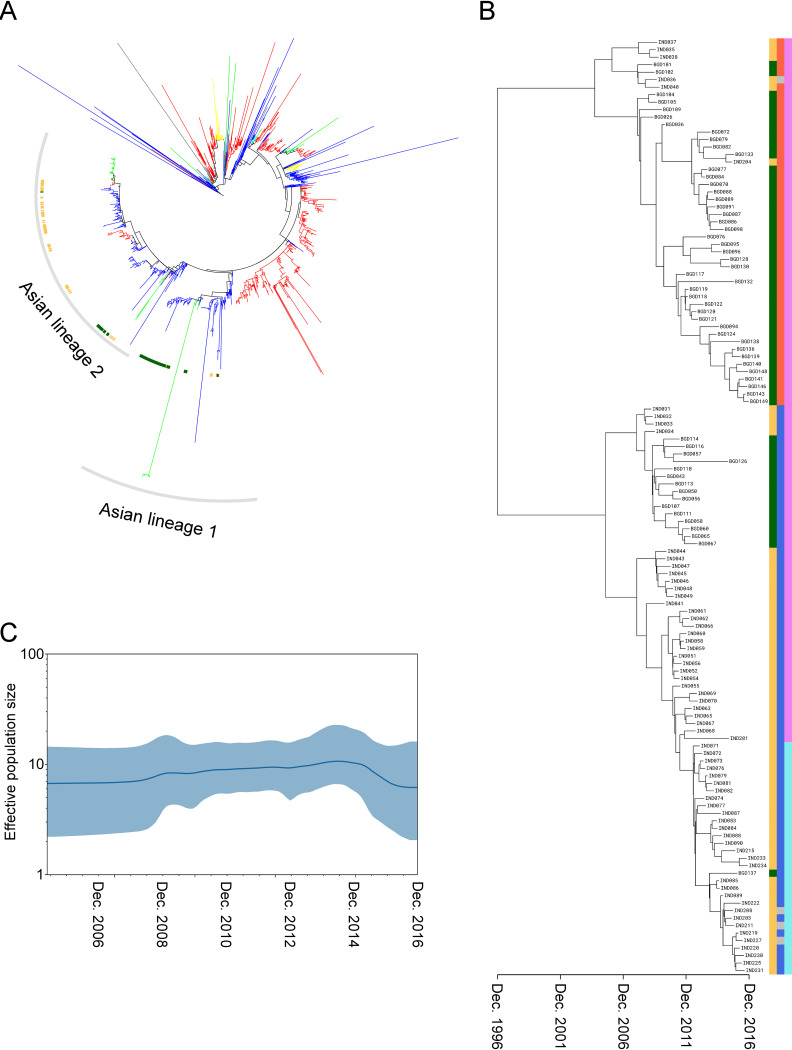
Phylogenetic and phylodynamic analysis of V. cholerae O1. (A) Maximum-likelihood phylogeny was determined using core genome alignment. Overall, 7,451 SNVs were identified in 1,303 strains; M66-2 was used as the outgroup. Branch color indicates the geographical region of origin for each strain: Asia, blue; Latin America, yellow; Africa, red; Europe, cyan; Middle East, green; unknown, gray. Colored boxes on the outside tree indicate strains from Dhaka, Bangladesh (dark green), and Kolkata, India (saffron yellow). Asian lineages are indicated with an outside gray curve. (B) Maximum clade credibility tree of 59 strains from Dhaka, Bangladesh, and 66 strains from Kolkata, India. Colored bars show information regarding the country (Bangladesh, dark green; India, saffron yellow), *ctxB* type (*ctxB1*, red; *ctxB7*, blue; negative, gray), and *carR* type (wild-type *carR*, violet; *carR* G265A, cyan). (C) Effective population size of V. cholerae O1 in the Bengal region, June 2005 to December 2016. The blue line represents the median effective population size estimate, and the shaded area represents the 95% high posterior density (95% HPD) interval.

We placed the 125 isolated strains from Kolkata and Dhaka among a global collection of 1,178 V. cholerae isolates by phylogenetic analysis based on the core genome single nucleotide variations (SNVs) (see Tables S1 and S2 in the supplemental material). All strains except M66-2 as an outgroup belonged to the seventh pandemic El Tor clade (Fig. 1A), with recent South Asian strains being classified into two lineages, Asian lineage 1 and Asian lineage 2. Asian lineage 1 also contained isolates from Iran, Iraq, China, and Vietnam, and isolates in Asian lineage 2 were introduced into Africa, Haiti, and Yemen by different events. All the Bengali isolates in this study belonged to the wave 3 clade of the seventh pandemic El Tor and were located on Asian lineage 1 or 2. Comparison of the dominant lineages in each year revealed a different temporal shift between Kolkata and Dhaka (Fig. S1). In Kolkata, the proportions of the two lineages initially appeared to be substantially equal, whereas after 2010 almost all isolates belonged to lineage 2. In comparison, in Dhaka, the dominance of lineage 1 fluctuated, decreasing once each in 2010 and 2011 and then being restored. Therefore, we performed phylodynamic analysis of the isolates from Kolkata and Dhaka to trace the sequential genomic changes and elucidate the detailed population structure of V. cholerae O1.

10.1128/mBio.01227-20.1 TABLE S1 Characterization of V. cholerae O1 isolates in Bengal. Download Table S1, XLSX file, 0.02 MB. Copyright © 2020 Morita et al. 2020 Morita et al. This content is distributed under the terms of the Creative Commons Attribution 4.0 International license.

10.1128/mBio.01227-20.2 TABLE S2 List of genomes from public databases. Download Table S2, XLSX file, 0.03 MB. Copyright © 2020 Morita et al. 2020 Morita et al. This content is distributed under the terms of the Creative Commons Attribution 4.0 International license.

10.1128/mBio.01227-20.4 FIG S1 Fate of the dominant lineage in each region. The proportion of each lineage of isolated strains in every year is shown. Colored bars show information regarding the lineages (lineage 1, red; lineage 2, blue). Download FIG S1, PDF file, 0.1 MB. Copyright © 2020 Morita et al. 2020 Morita et al. This content is distributed under the terms of the Creative Commons Attribution 4.0 International license.

The maximum clade credibility tree also showed two distinct lineages that were concurrently distributed in Bengal. The dates of the most recent common ancestor of lineage 1 and lineage 2 were estimated as August 2004 (95% high posterior density [95% HPD], October 2000 to October 2007) and June 2005 (95% HPD, September 2001 to June 2008), respectively (Fig. 1B). Within lineage 1, isolates circulating among the two regions in 2009 formed a different sublineage from another sublineage that persisted in Dhaka. Similarly, lineage 2 diverged into two sublineages, with one sublineage consisting mainly of Kolkata isolates and the other transient sublineage observed until April 2015. It appeared that in both regions, a specific lineage may have adapted to the environmental conditions with continuous modification of the genome and became dominant. In contrast, other sublineages disappeared. Therefore, the effective population size of V. cholerae O1 significantly decreased after 2015 (Fig. 1C).

### Genetic characterization of *ctxB*, *rtxA*, *carR*, and VSP-II.

Phylogenetic analysis revealed that the Bengali strains could be classified into two lineages, with each lineage possessing different genetic traits (Fig. 1B; Table S1). Each lineage was associated with a *ctxB* genotype except for four *ctxB*-negative strains; specifically, isolates in lineage 1 contained *ctxB1*, and isolates in lineage 2 contained *ctxB7*. Further, there was a G-to-A mutation at position 13602 in *rtxA* of lineage 2, which generated a stop codon. Recently polymyxin-susceptible strains were reported among the El Tor biotype owing to an SNV in the *carR* gene (G265A; resulting in CarR D89N) (20, 21). An isolate in lineage 2 containing mutant *carR* was first detected in January 2013, and acquisition of its SNV was estimated as occurring in June 2012, consistent with prior estimates (12). These isolates formed a specific cluster and were maintained in the population.

VSP-II was identified to be unique in the seventh pandemic El Tor strains, although the role of the genomic islands (GIs) remains to be established (22). VSP-II constitutes a 26.9-kb genomic region composed of 24 open reading frames (ORFs) between VC0490 and VC0516 according to the annotation of V. cholerae N16961. Previous studies, including those by our laboratory, demonstrated diversity of VSP-II across the strains (18, 23). In particular, we found that all strains partially lacked VSP-II and could be divided into five types: those with a deletion from VC0495 to VC0512 (type 1), those with a deletion from VC0495 to VC0498 (type 2), those with a deletion from VC0495 to VC0498 and a frameshift of VC0490 (type 3), those with a deletion from VC0492 to VC0498 and a frameshift of VC0490 (type 4), and those negative for VSP-II (type 5) (Fig. S2). All strains in lineage 2 possessed type 1 VSP-II. In contrast, lineage 1 contained four types of VSP-II, including type 2 to type 5. The transient sublineage detected in 2009 contained strains carrying type 2 VSP-II and one VSP-II-negative strain (type 5). Subsequently, the major type of VSP-II in lineage 1 gradually shifted from type 3 to type 4 (Table S3). The entire deletion of VSP-II would be caused by excision of the VSP-II locus (type 5), with modification from type 2 to type 4 occurring through stepwise VSP-II deletion.

10.1128/mBio.01227-20.3 TABLE S3 Chronological diversity of VSP-II types. Download Table S3, XLSX file, 0.01 MB. Copyright © 2020 Morita et al. 2020 Morita et al. This content is distributed under the terms of the Creative Commons Attribution 4.0 International license.

10.1128/mBio.01227-20.5 FIG S2 Schematic diagram of VSP-II. The transcription direction of each gene is indicated by an arrow, and each type of region is differentially shaded. The VC0490 frameshift in type 2 and 3 is indicated by a broken arrow. Download FIG S2, PDF file, 0.4 MB. Copyright © 2020 Morita et al. 2020 Morita et al. This content is distributed under the terms of the Creative Commons Attribution 4.0 International license.

In previous reports, partial sequences of VSP-II were found in the non-O1, non-O139 V. cholerae genome at sites other than the original integration site (23, 24). Among 125 Bengali isolates, only four strains of lineage 2 (BGD101, BGD102, IND036, and IND040) possessed the 18.2-kb GI at the site between VC0153 and VC0154, which shared the 11 ORFs of VSP-II (Fig. S3). The sequence revealed 97% similarity with Vibrio anguillarum VIB43 (CK207_00770 to CK207_00865 of DDBJ/EMBL/GenBank accession number CP023054) (25). This thus represents the first report of the discovery of the novel GI at VC0153 to VC0154 in V. cholerae O1 strains.

10.1128/mBio.01227-20.6 FIG S3 Schematic diagram of the novel GI at VC0153 to VC0154 in Bengali V. cholerae O1 and VSP-II in V. cholerae O1 N16961. The transcription direction of each gene is indicated by an arrow, and homologous regions are shaded. Download FIG S3, PDF file, 0.2 MB. Copyright © 2020 Morita et al. 2020 Morita et al. This content is distributed under the terms of the Creative Commons Attribution 4.0 International license.

### Antimicrobial resistance-related genotype analysis.

Multiantimicrobial resistance constitutes a major characteristic of recent pathogenic V. cholerae O1 isolates, with ICE of the SXT/R391 family containing determinants of the resistance phenotype in V. cholerae O1 of the seventh cholera pandemic (9). ICE is arranged in a mosaic genetic structure composed of a conserved core region and interspersed variable sequences located in specific sites. The conserved core region comprises 52 genes, of which 25 are related to the function of conjugative transfer. The interspersed variable sequences are located in specific sites termed hot spots (HS1 to -5) and variable regions (vI to vIV). The variable sequences often contain environmental adaptive genes, such as those coding for antimicrobial resistance and restriction and modification systems. As the core region is highly conserved, variation in SXT/R391 ICEs was defined by the variable sequences (26). Accordingly, to investigate the determinant of antimicrobial resistance, we determined the structure of ICEs and detected antimicrobial resistance genes (Fig. S4). ICEs in the strains were also divided into two types, corresponding with the two lineages (Table S1). The ICE in lineage 1 contained antimicrobial resistance genes *sul2*, *dfrA*, *tetA*(D), and *strAB*. Because ICEs contain several similar transposases, the sequence of the ICE was split into several contigs. The ICE in lineage 1 appeared almost identical to that found in V. cholerae ICDC-2255 isolated in China (ICE*Vch*Chn2255) (27). In turn, another ICE in lineage 2 contained antimicrobial resistance genes *sul2*, *dfrA*, *floR*, and *strAB*. This ICE also revealed almost complete identity to ICE*Vch*Ind5 (26). In addition, the ICE of one isolate in lineage 1 lacked the vIII region containing *sul2*, *tetA*(D), and *strAB*. Furthermore, 10 isolates in lineage 2 lacked the ICE region from the vIII region to *xis*, comprising *sul2*, *floR*, and *strAB*. Based on the phylogenetic tree, these 10 isolates occupied the same branch and transiently appeared. As these antimicrobial resistance genes are flanked by transposase genes, this deletion has often been reported (12, 18, 28).

10.1128/mBio.01227-20.7 FIG S4 Structure of SXT/R391 family ICEs and accessory gene organization. SXT/R391 family ICEs are classified by inserted regions, termed hot spots 1 to 5 (HS1 to -5) and variable regions (vI to vIV). Similar sequence regions are indicated by the same color. Locations of antibiotic resistance genes are also represented (Tc, tetracycline resistance; Cm, chloramphenicol resistance; Sm, streptomycin resistance; Su, sulfamethoxazole resistance; Tm, trimethoprim resistance). Download FIG S4, PDF file, 0.1 MB. Copyright © 2020 Morita et al. 2020 Morita et al. This content is distributed under the terms of the Creative Commons Attribution 4.0 International license.

The antimicrobial resistance genes in two types of ICE were almost the same; they included *sul2*, *dfrA*, and *strAB* but not *floR* and *tetA*(D). Therefore, in addition to their common antimicrobial resistance against streptomycin and SXT, lineage 1 is expected to be resistant to tetracyclines and lineage 2 to chloramphenicol.

Spontaneous mutations in quinolone resistance-determining regions (QRDRs) of the DNA gyrase genes *gyrAB* and topoisomerase IV genes *parCE* also serve as important antimicrobial resistance factors in V. cholerae, as fluoroquinolones are also used for cholera treatment (12, 24, 29, 30). We detected mutations in *parC* that resulted in an S85L substitution and mutations in *gyrA* that resulted in an S83I amino acid substitution in all strains in both lineages. Furthermore, the strains in lineage 1, except for BGD101, BGD102, IND035, IND036, IND037, IND038, and IND040, contained an additional *gyrA* mutation that resulted in D660E. However, the mutation GyrA D660E is not reported as being within a QRDR and would not be expected to be associated with quinolone resistance. Consistent with their sequences, these seven strains were located in the same branch of the phylogenetic tree.

## DISCUSSION

We performed genome-wide sequencing on 125 Bengali V. cholerae O1 isolates (59 strains from Kolkata, India, and 66 from Dhaka, Bangladesh) obtained from 2009 to 2016 and conducted core genome analysis in conjunction with a global collection of 1,178 V. cholerae isolates. Bengali isolates in this study were related to two clades of global phylogeny, Asian lineage 1 and Asian lineage 2. The relationship between Bengali strains and globally prevalent strains supports the origin of the V. cholerae O1 that spread to other countries in the Bay of Bengal region (9). Lineage 1 and lineage 2 occupied the largest proportion of isolates from Dhaka and Kolkata, respectively. In each region, isolates in another lineage appeared transiently and specific sublineages were maintained. This phenomenon resulted in the decrease of genetic diversity in each region and indicated that each lineage likely adapted to the environmental condition in each region.

Notably, each lineage exhibited different *ctxB* genotypes. Isolates in lineage 1 harbored *ctxB1*, and isolates in lineage 2 carried *ctxB7*. The genotypes of *ctxB* were related to the clade of the seventh pandemic El Tor: *ctxB3* in wave 1, *ctxB1* in wave 2 and early wave 3, and *ctxB7* in the current wave 3 clade (9). All the isolates in the present study belonged to the wave 3 clade of the seventh pandemic El Tor based on core genome analysis. Therefore, lineage 1 and lineage 2 may be correlated with early wave 3 and current wave 3, respectively. This result indicated that early wave 3 has remained in Bangladesh. In comparison, the isolates in the recent catastrophic outbreaks in Yemen and Haiti were related to lineage 2, and several reports demonstrated that the source of spreading was derived from India (12, 19), whereas lineage 1 has been associated with epidemics in China and Vietnam (27, 31). The factor(s) causing the difference in epidemic scale was unclear. However, different geographical situations may influence the source of the epidemic in various countries.

Moreover, each lineage differed not only in the core genome but also in the genomic islands VSP-II and ICE. The prototypical VSP-II was discovered in 2004 and encoded transcriptional regulators, methyl-accepting chemotaxis proteins, a putative RNase, a putative type IV pilin, a DNA repair protein, an integrase, and a number of hypothetical proteins. Although several variants have been identified from different continents, the role of VSP-II remains unclear (9, 13–17, 23). ICEs constitute genetic elements transferable by conjugation and confer multiantimicrobial resistance. ICEs have been detected since the emergence of wave 2, and several variants have been reported worldwide. As antimicrobial resistance plasmids have rarely been reported in V. cholerae O1 after wave 2, ICEs are therefore currently considered the main factor contributing to antimicrobial resistance in V. cholerae O1 (9, 26).

In the present study, VSP-II variants in each lineage exhibited different trends. In lineage 1, four types of VSP-II variants were identified, from type 2 to type 5 (see Fig. S2 in the supplemental material). The VSP-II variants changed from type 2 to type 4, being deleted stepwise over time. In contrast, the VSP-II variant in lineage 2 was identified as type 1 only. In turn, ICEs confer antimicrobial resistances in bacteria, and all strains, except one Bangladesh isolate, carried ICEs. The ICEs were almost identical to the reported ICEs ICDC-2255 in lineage 1 and ICE*Vch*Ind5 in lineage 2. In addition, 1 and 10 ICEs in lineage 1 and lineage 2, respectively, lacked a part of the region containing antimicrobial resistance genes, and these strains rapidly disappeared over time. As antibiotic therapy provides huge selective pressure, these strains might not be compatible with the clinical situation. Furthermore, a portion of the antimicrobial resistance pattern provided by ICEs differed in lineage 1 and lineage 2, which carried the tetracycline resistance gene *tetA*(D) and the chloramphenicol resistance gene *floR*, respectively. The difference in VSP-II variants and ICEs might have reflected the fitness of each lineage in the unique environmental conditions in India and Bangladesh, which would result in the existence of two lineages in the Bengal region.

The outbreaks in Haiti and Yemen were triggered by high-risk situations due to the breakdown of infrastructure facilities as a result of earthquake and civil war. It has also been suggested that the lack of preexisting immunity owing to the absence of cholera for 100 years in Haiti might have contributed to instigating the outbreak (2). Therefore, it is anticipated that the lineage 1 clade might also induce outbreaks in any part of the world under similar situations. In such a case, a difference of resistance in each lineage would constitute a serious problem. From the viewpoint of genetic analysis, although almost a full set of resistance genes associated with the new quinolones streptomycin and SXT was common in each lineage, each lineage also carried a different resistance gene associated with tetracycline resistance (lineage 1) or chloramphenicol resistance (lineage 2). Notably, these drugs are also used for cholera treatment, with the tetracycline derivative (doxycycline) especially utilized as a first-line drug (32). However, doxycycline would be less effective for lineage 1. When the infectious agents in lineage 1 spread globally, the use of doxycycline facilitates the replacement of lineage 2 with lineage 1 because doxycycline might fail to prevent lineage 1. Therefore, to provide appropriate treatment, it is important to ascertain whether the infectious agent belongs to lineage 1 or lineage 2.

We consider that surveillance of V. cholerae in Bengal is therefore critical to correctly predict the risk of global cholera spread. We also posit that a better understanding of cholera and the ability to curb this disease can be achieved through genomics. Ultimately, these evaluations will help to build a more complete picture of the global spread and dynamics of this fatal disease.

## MATERIALS AND METHODS

### Bacterial isolates and whole-genome sequencing.

We collected 59 V. cholerae O1 isolates from Dhaka, Bangladesh, and 66 from Kolkata, India (see Table S1 in the supplemental material). The hospitals where isolates originated were icddr,b Dhaka Hospital in Dhaka, Infectious Diseases and Beliaghata General Hospital in Kolkata, and Dr. B C Roy Post Graduate Institute of Pediatric Sciences in Kolkata. A total of 51 strains of Kolkata isolates had already been sequenced in our previous study (17). All isolates were obtained from fecal samples of hospitalized patients with typical cholera symptoms. V. cholerae was isolated by streaking the stool samples on thiosulfate citrate bile salts sucrose (TCBS) agar plates, and typical sucrose-fermenting yellow colonies were considered presumptive V. cholerae. Single isolated yellow colonies were streaked on an LB agar plate (Difco), and then the bacterial lawn on the LB agar plate was subjected to an oxidase and string test as described earlier (31). Oxidase and string test-positive cultures were checked for slide agglutination to confirm their serogroup with polyvalent O1-specific antisera according to the manufacturer’s instruction (Denka Seiken). Genomic DNA was prepared using an DNeasy blood and tissue kit (Qiagen) according to the manufacturer’s instructions. We prepared Illumina libraries using the Nextera XT DNA library preparation kit (Illumina) and sequenced each library on a MiSeq (Illumina) sequencer as 2 × 300 bp paired-end reads. Nucleotide sequence data were submitted to the DDBJ Sequenced Read Archive, and each accession number is given below, as well as in Table S1.

### Phylogenetic and phylodynamic reconstruction.

To perform phylogenetic analysis, 1,178 genome sequences were obtained from the public database, and M66-2 was used as the outgroup (Table S2). Genome assembly was performed using SPAdes version 3.13.1 with default parameters (33). Variants were called using Snippy version 4.3.6 (https://github.com/tseemann/snippy) using the V. cholerae O1 El Tor N16961 genome (GenBank accession numbers LT907989 and LT907990) as a reference. We excluded the SNVs in recombinogenic regions detected using Gubbins version 2.3.4 (34), along with the SNVs in the repeat and prophage regions of the N16961 genome, which were identified using NUCmer (35) and PHAST (36) for core genome phylogeny. A maximum likelihood phylogenetic tree was generated using RAxML version 8.2.0 (37) with 1,000 bootstrap iterations.

To estimate the phylodynamics of the V. cholerae O1 isolates in the Bengal region, we performed a temporal analysis using the Bayesian Evolutionary Analysis Sampling Trees (BEAST) version 2.4.7 software package (38). A relaxed clock exponential model and an HKY nucleotide substitution model were implemented using the Markov chain Monte Carlo method run for 100 million generations and sampled every 1,000 generations. The effective sample sizes were >200 for all estimated parameters. The tree data were summarized to generate the maximum clade credibility tree using TreeAnnotator with first 10% burn-in. The effective population size was reconstructed using the Bayesian coalescent skyline plot implemented in Tracer version 1.6.0. These results were visualized using Interactive Tree Of Life (iTOL) version 4 (https://itol.embl.de) (39).

### Identification of VSP-II and novel genomic islands.

The contigs of the draft genome from each isolate were aligned against the VSP-II region (VC0489 to VC0517 of N16961) using BLAST+ version 2.6.0 to identify the deletion of VSP-II (40). The sequence from VC0153 to VC0154 was extracted from the draft genome sequence of each strain using BLAST+ version 2.6.0 and an in-house Perl script to elucidate whether the genetic element was inserted at this locus.

### Identification of ICE.

The contigs containing the conserved core region of ICE were selected using BLAST+ version 2.6.0, and sequence comparisons were performed with MAUVE (41). Sequences of ICE*Vch*Ind5 and ICE in ICE*Vch*Chn2255 (GenBank accession numbers GQ463142 and KT151660) were used as references. One strain (BGD138) was found to lack the entire ICE sequence, as no genetic element was located at the ICE insertion sites VC0658 and VC0659.

### Data availability.

Nucleotide sequence data generated in this study are available in the DDBJ/EMBL/GenBank databases under accession numbers DRX179739 to DRX179812.
