# Supplementary figures and images for: Whole-Genome Analysis of Clinical Vibrio cholerae O1 in Kolkata, India, and Dhaka, Bangladesh, Reveals Two Lineages of Circulating Strains, Indicating Variation in Genomic Attributes
Source: mBio. 2020 Nov 10;11(6):e01227-20. doi: 10.1128/mBio.01227-20 (PMC7667034; doi:10.1128/mBio.01227-20)

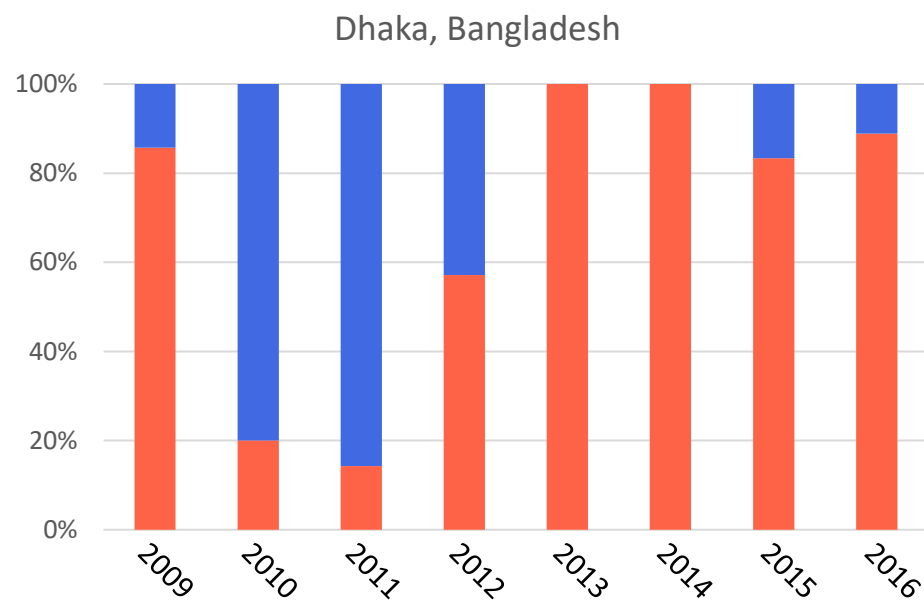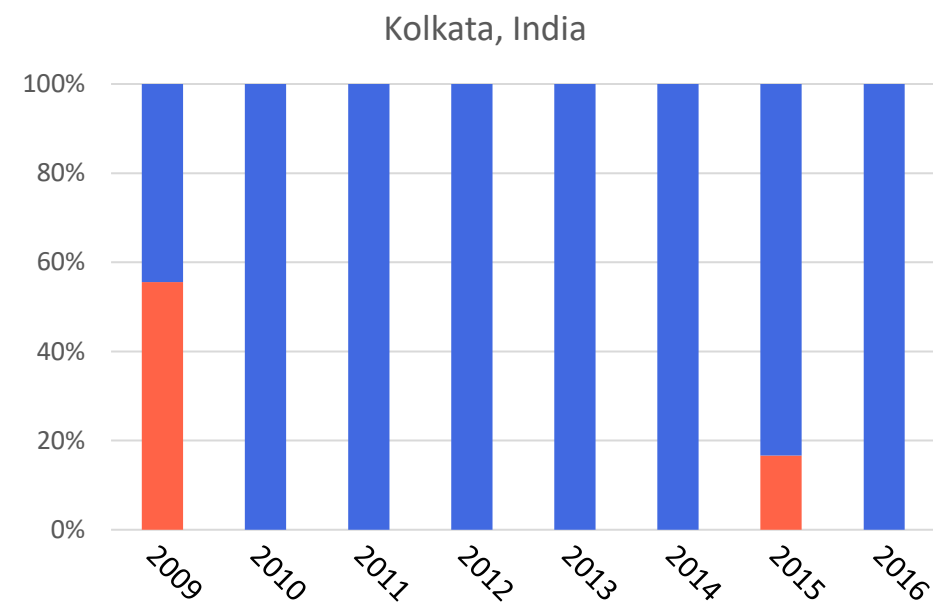

Fig S1

Supplement: FIG S1 [file mBio.01227-20-sf001.pdf]

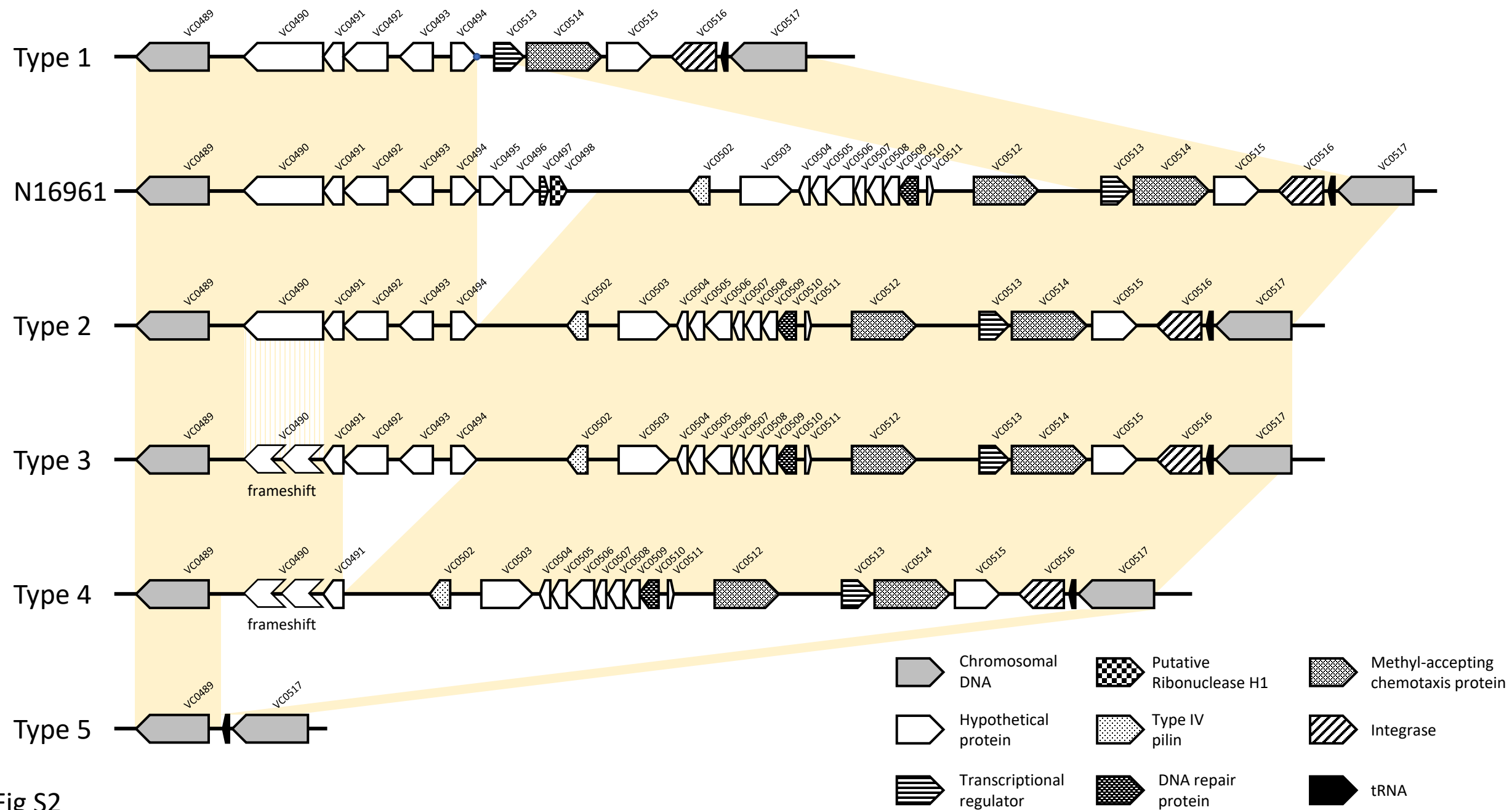

Fig S2

Supplement: FIG S2 [file mBio.01227-20-sf002.pdf]

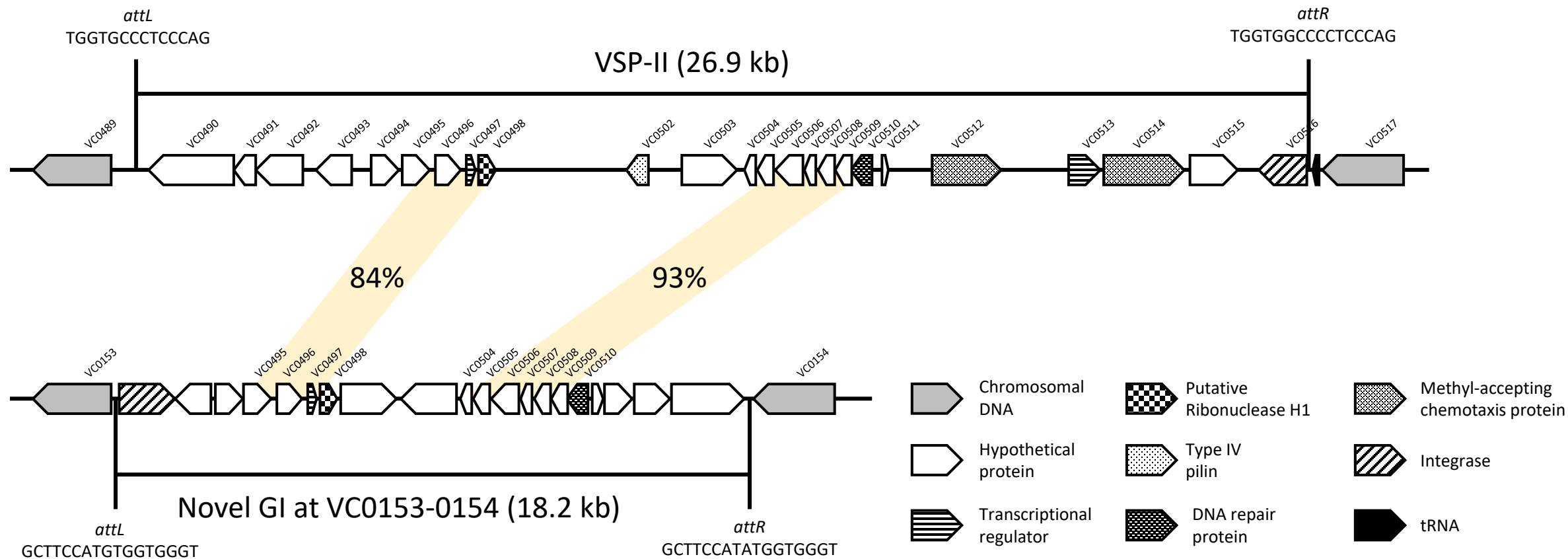

Fig S3

Supplement: FIG S3 [file mBio.01227-20-sf003.pdf]

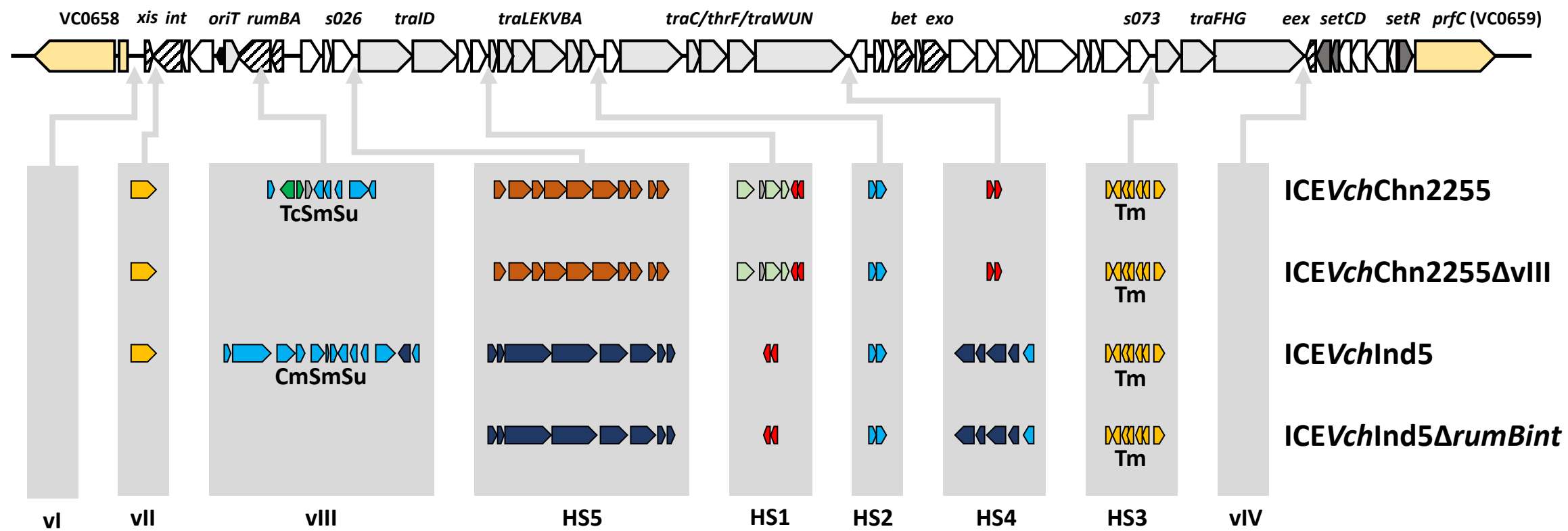

Fig S4

Supplement: FIG S4 [file mBio.01227-20-sf004.pdf]
